# What have we learned from cancer immunotherapy in the last 3 years?

**DOI:** 10.1186/1479-5876-12-141

**Published:** 2014-05-21

**Authors:** Paolo A Ascierto, Francesco M Marincola

**Affiliations:** 1 Unit of Melanoma, Cancer Immunotherapy and Innovative Therapy Unit, Istituto Nazionale Tumori Fondazione “G. Pascale”, Via Mariano Semmola, 80131 Naples, Italy; 2 Sidra Medical and Research Centre, Doha, Qatar

**Keywords:** Immunotherapy, Melanoma, Efficacy, Survival, Sequencing

## Abstract

Until recently, most immunotherapeutic approaches used to fight cancer were ineffective, counteracted by the tumour’s ability to evade immune attack. However, extensive research has improved our understanding of tumour immunology and enabled the development of novel treatments that can harness the patient’s immune system and prevent immune escape. Over the last few years, through numerous clinical trials and real-world experience, we have accumulated a large amount of evidence regarding the potential for long-term survival with immunotherapy agents in various types of malignancy. The results of these studies have also highlighted a number of recurring observations with immuno-oncology agents, including their potential for clinical application across a broad patient population and for both conventional and unconventional response patterns. Furthermore, given the numerous immune checkpoints that exist and the multiple mechanisms used by tumours to escape the immune system, targeting distinct checkpoint pathways using combination approaches is an attractive therapeutic strategy with the potential to further enhance the antitumour immune response.

## Background

Exploiting the immune system’s ability to identify and destroy tumours using immunotherapy has long been recognised as a promising approach to anticancer treatment [1]. However, traditional immunotherapies such as interferon and interleukin-2 have generally failed to demonstrate consistent clinical benefit in advanced stage cancer.

The recent renaissance of cancer immunotherapy can be largely attributed to the development of novel immunotherapy agents which target specific immune regulatory checkpoints to enhance the endogenous antitumour immune response. After becoming the first agent to demonstrate a significant overall survival (OS) improvement in a randomised phase 3 trial in metastatic melanoma [2], the anti-cytotoxic T-lymphocyte antigen-4 (CTLA-4) antibody ipilimumab was approved for this indication (although, in Europe, its initial indication was restricted to patients who had received prior therapy) [3]. In the last 3 years, we have gained a wealth of experience with this and other immunotherapies in the clinical setting and learned a considerable amount regarding their potential benefit across multiple tumour types.

CTLA-4 is a key inhibitory checkpoint molecule that is thought to counteract the co-stimulatory signal from its homologue, CD28, by competitively binding to its ligands (B7.1 and B7.2) on the surface of antigen-presenting cells [4]. Tumour cells exploit this pathway to turn off the immune response by suppressing the activation and proliferation of conventional T cells and promoting the function of regulatory T cells (Tregs) which can dampen the immune response at the tumour site [5,6]. By blocking CTLA-4, ipilimumab restores the co-stimulatory activity of CD28, thereby increasing the number of activated T cells that can migrate to and attack the tumour [7]. Recent data have also demonstrated that treatment with CTLA-4 antibodies can mediate selective depletion of Treg cells within the tumour [8].

**Figure 1:**
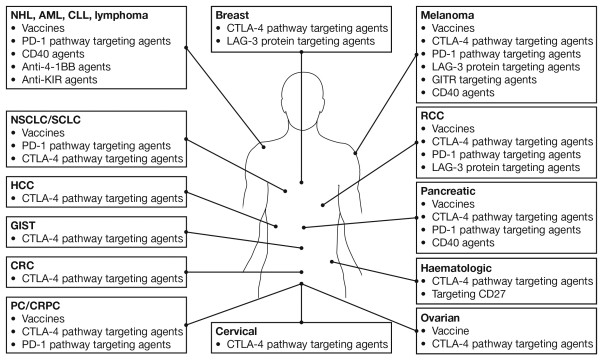
**Immuno-oncology agents**^**a**^** in clinical development across multiple tumour types.**^a^Selected therapies and tumour types are shown: additional agents are, for example in phase 1 studies in patients with solid tumours [12]. AML, acute myeloid leukemia; CLL, chronic lymphocytic leukaemia; CRC, colorectal cancer; CRPC, castration-resistant prostate cancer; CTLA-4, cytotoxic T-lymphocyte antigen-4; GIST, gastrointestinal stromal tumour; HCC, hepatic cell carcinoma; LAG-3, lymphocyte activation gene 3; mAb, monoclonal antibody; NHL, non-Hodgkin lymphoma; NSCLC, non-small cell lung cancer; PC, prostate cancer; PD1, programmed death 1; RCC, renal cell carcinoma; SCLC, small cell lung cancer.

In addition to CTLA-4, numerous other immune checkpoints exist that are potential targets for immunotherapy [9,10]. For example, interaction of the programmed death 1 (PD1) receptor with its ligands (PDL1 and PDL2) in peripheral sites leads to T-cell inactivation and loss of effector function. Targeting this pathway using antibodies against PD1 (e.g. nivolumab, pembrolizumab) or PDL1/PDL2 (e.g. MPDL3280A, MEDI4736) breaks down this mechanism, preventing T-cell inactivation and restoring immune activity directly at the tumour site [11]. Immunotherapies targeting other immune checkpoint molecules such as LAG3 and CD137 (4-1-BB) are also under evaluation in advanced malignancies, either as monotherapy or in combination with other therapies (Figure 1).

Adoptive T cell therapy (ATC) using chimeric antigen receptors (CARs) is an alternative immunotherapeutic approach to anticancer therapy. This approach combines the antigen-binding property of monoclonal antibodies with the lytic capacity and self-renewal of T cells [13]. Clinical trials have revealed promising results in patients with CD19-positive haematological malignancies, including non-Hodgkin’s lymphoma, chronic lymphocytic leukaemia, and acute lymphoblastic leukaemia; further trials in patients with B-cell malignancies or solid tumours are ongoing [13].

ATC therapy using autologous *ex vivo-*expanded tumour infiltrating lymphocytes (TILs) that are then adoptively transferred back into patients is an immunotherapy that has shown clinical efficacy in metastatic melanoma [14]. In one study, the immunodominant epitope recognised by the tumour-reactive T-cells was identified as a mutated protein phosphatase 1, regulatory (inhibitor) subunit 3B (PPP1R3B) gene product; the patient achieved a durable complete response with regression of bulky liver tumour mass [15].

Efforts to determine an effective vaccine that alerts the immune system to cancer cells have largely failed; however, experimental cancer vaccines containing proteins that are overexpressed by tumour cells may work synergistically with other immunotherapies [16,17]. Talimogene laherparepvec (T-VEC) is a genetically modified virus which drives the secretion of the immunostimulatory cytokine granulocyte macrophage colony stimulating factor (GM-CSF) [18]. T-VEC is currently being evaluated as a potential treatment in melanoma and other advanced cancers [19,20]. In a pre-clinical study in breast cancer bearing mice, the combination of an anti-PD1 antibody and a multi-peptide vaccine prolonged the vaccine-induced progression-free survival (PFS) by altering both the CD8 T-cell and dendritic cell components of the tumour microenvironment [21].

Currently, the American Joint Committee on Cancer (AJCC)/International Union Against Cancer (UICC) tumor-node-metastasis (TNM) classification provides limited prognostic information and does not predict response to therapy. However, it is now becoming clear that the host-immune reaction to tumours is a critical element in determining response to therapy. This has led to the development of an ‘Immunoscore’ which correlates immune-cell infiltration in tumors to patient’s clinical outcome. Recent work has reported that such an immune-classification has a prognostic value that may be superior to the AJCC/UICC TNM-classification, and studies are ongoing to validate and integrate such a system into clinical practice [22].

Responses to immunotherapy may be delayed or may develop after a period of apparent disease progression due to the time required to build an effective immune response; therefore, tumour assessments should be performed only after completion of the assigned regimen and the results confirmed with a follow-up scan [23]. For ipilimumab, the recommended induction regimen is 3 mg/kg, administered every 3 weeks for 4 doses; this is sufficient to provide a considerable survival benefit in a proportion of patients [2,3]. To maximise clinical benefit, it is also recommended that ipilimumab is administered for the entire induction regimen as tolerated, regardless of the appearance of new lesions or growth of existing lesions, as immune cell infiltration following immunotherapy may be mistaken for tumour progression [24]. In addition, the unconventional nature of some responses with ipilimumab have made it necessary to introduce new criteria to characterise antitumour activity, as conventional assessment methods may not fully capture these novel response patterns. Immune-related response criteria (irRC) were developed from existing modified World Health Organisation (mWHO) criteria; the irRC allow for initial tumour progression or appearence of new lesions, both of which would be considered as progressive disease according to mWHO criteria [23]. Although irRC were developed based on response patterns observed with ipilimumab, the possibility of unconventional, immune-related response patterns must be also considered for other immunotherapies, including nivolumab and other anti-PD1/PDL1 agents. Indeed, responses with nivolumab may be rapid or delayed, and may continue after therapy is discontinued [25].

An emerging understanding of the biology affecting the results of immunotherapy leads to the conclusion that surrogate endpoints such as objective response rates and PFS may not be appropriate for measuring long-term treatment benefit [26]. Data from clinical trials and ipilimumab expanded access programmes (EAPs) indicate that long-lasting stable disease is a common outcome with immunotherapy and that, even in the absence of a complete or partial tumour response, durable disease control can result in prolonged OS [23,27]. Efficacy endpoints that better correlate with prolonged survival, such as landmark survival analyses are therefore becoming more relevant since they take into consideration the durability of survival [26].

**Figure 2:**
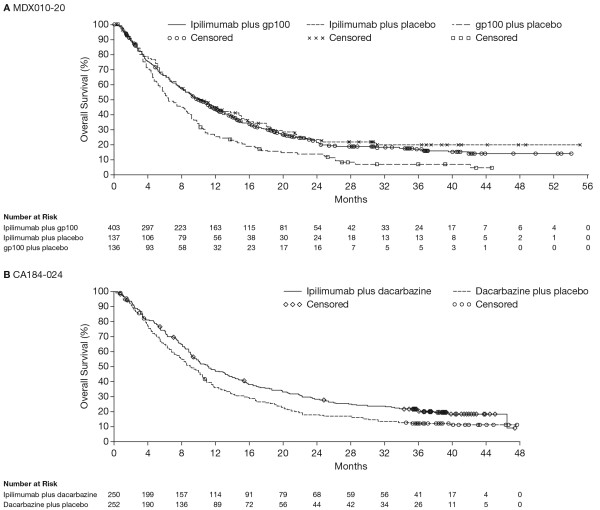
**Kaplan-Meier curves of OS in patients treated with ipilimumab.** OS curves from two randomised phase 3 trials of ipilimumab in patients with metastatic melanoma: **A)** MDX010-20 trial, and **B)** CA184-024 trial.

Kaplan-Meier curves of OS in patients treated with ipilimumab show that survival consistently reaches a plateau at around 2–3 years [2,28-31], as demonstrated in both randomised phase 3 trials of ipilimumab in metastatic melanoma (Figure 2). Beyond this time point, the long ‘tail’ of the survival curve reflects the emergence of long-term survivors and highlights the importance of using landmark survival analyses as well as hazard ratios and median OS, to benchmark survival outcomes. In a phase 3 trial investigating survival with ipilimumab in patients with metastatic castration-resistant prostate cancer (mCRPC), the 2-year survival rate with ipilimumab was 26%, compared with 15% for patients in the placebo arm [32]. Furthermore, in a recent meta-analysis of 1,861 patients with metastatic melanoma who received ipilimumab in phase 2 and phase 3 trials, the 3-year survival rate was 22% and around one fifth of patients survived for up to 10 years, irrespective of prior treatment [33]. Notably, inclusion of EAP data did not affect the overall shape of the pooled survival curve, indicating that the potential for long-term survival persists even in patients with a particularly poor prognosis. In both the meta-analysis and the prostate cancer trial, the shape of the OS curves for ipilimumab-treated patients appeared remarkably similar to those seen in the pivotal phase 3 trials in melanoma. Additional data from clinical trials suggest that targeting other immune checkpoints can provide long-term survival benefits. For example, in a phase 1 clinical trial evaluating nivolumab in patients with advanced solid tumours, the 2-year survival rates were 14% in non-small cell lung cancer (NSCLC), 43% in melanoma, and 50% in renal cell carcinoma (RCC) [34,35]. At present, no median OS or long-term survival data are available for pembrolizumab; however, rapid and durable tumour regressions have been observed with this agent as well as with nivolumab. Indeed, durable objective responses may be considered a key endpoint in the future as we enter a new era of immunotherapy requiring more stringent evaluation [36]. With regard to other immunotherapies, in an interim survival analysis from a phase 3 trial of T-VEC versus GM-CSF in patients with advanced melanoma, the 3-year survival rates were 41% and 28% for patients treated with T-VEC and GM-CSF, respectively [19,20]. These results are extremely encouraging for patients with advanced malignancies and have fuelled speculation that the availability of novel immunotherapies could potentially result in cancer turning into a controllable chronic disease in a considerable proportion of patients.

Evidence suggests that a number of immuno-oncology approaches do not require identification of specific tumour antigens and thus provide the potential to offer clinical benefit across many different types of cancer. Based on this rationale, monoclonal antibodies targeting a variety of immune checkpoints inhibitors (e.g. anti-CTLA-4, anti-PD1, and anti-LAG3) have been investigated across multiple tumour types, including prostate cancer, lung cancer and RCC [32,34,37-42]. For example, in a phase 3 trial in patients with mCRPC, ipilimumab treatment improved PFS and prostate specific antigen (PSA) responses versus placebo when administered after a single dose of radiotherapy (RT). Although the primary endpoint of improved OS was not met (the hazard ratio for OS versus placebo was 0.85; *P* = 0.05), the results of an OS subgroup analysis suggested that patients with a lower disease burden may be more likely to benefit from ipilimumab treatment [32]; an ongoing phase 3 trial of ipilimumab in patients with chemotherapy-naïve mCRPC is prospectively evaluating this patient population.

Encouraging results have also been observed in ipilimumab clinical trials in patients with advanced lung cancer. In a phase 2 trial, there was a trend towards improved survival in patients who received ipilimumab 10 mg/kg after carboplatin and paclitaxel (CP) compared with CP alone [38,39]; two randomised phase 3 trials to evaluate ipilimumab in NSCLC (NCT01285609) or SCLC (NCT01450761) are open for enrollment.

Among other investigational immunotherapies, the anti-PD1 antibody nivolumab has been evaluated in a variety of tumour types. In the phase 1 trial described earlier, median OS was 9.6 months, 16.8 months and >22 months, in patients with NSCLC, melanoma and RCC, respectively [34,35]; phase 3 trials of nivolumab in each of these indications are currently ongoing. Other immune checkpoint inhibitors, including other anti-PD1/PDL1 agents, anti-LAG3 antibodies and anti-KIR antibodies, are also under evaluation in various solid tumours and haematological malignancies.

As immunotherapy works to enhance the host’s own immune system rather that acting directly on the tumour itself, immuno-oncology approaches have the potential to be effective across patient subpopulations, regardless of mutational status (e.g. BRAF/NRAS) or histological subtype. In melanoma, BRAF inhibitors (vemurafenib and dabrafenib) can provide rapid responses in the 40–50% of patients with a mutation in BRAF V600; however, their use is contra-indicated in patients with wild-type BRAF status [43-45]. By contrast, BRAF or NRAS mutation status does not appear to be associated with the clinical activity of ipilimumab (Table 1). In a retrospective multicentre analysis, there was no difference in median OS between patients with BRAF/NRAS-mutated or wild-type melanoma (10.12 months versus 10.18 months) treated with anti-CTLA-4 antibodies [46]. Similarly, disease control rates and survival were comparable between BRAF/NRAS mutant and wild-type patients in the Italian EAP; safety results were consistent across all groups with respect to mutational status [47].

**Table 1:** Clinical trial and real-world data on the use of ipilimumab in patient subpopulations

**Patient subgroup**	**Efficacy summary**	**Safety summary**	**References**
Elderly patients	*Italian EAP ( >70 years)*		
	DCR: 38%	Generally well tolerated; consistent with wider EAP	Chiarion Sileni et al., 2014 [48]
	1- year OS: 38%		
	2-year OS: 22%	population	
	*Spanish EAP (≥65 years)*		
	DCR: 35%	No increase in toxicity in elderly patients	Lopez Martin et al., 2012 [49]
	1- year OS: 21%		
	*US EAP (≥65 years)*	Consistent with wider EAP population	Lawrence et al., 2012 [50,51]
	1- year OS: 37%		
	*NYU retrospective analysis*		Chandra et al., 2013 [52]
	*(≥65 years)*	Consistent with published data in younger cohorts	
	DCR: 36%		
Uveal melanoma	*Italian EAP*		
	DCR: 34%	Safety profile similar to that in cutaneous melanoma	Maio et al., 2013 [53]
	1- year OS: 31%		
	*I-OMEAP (10 mg/kg)*	Consistent with ipilimumab clinical trials	Danielli et al., 2012 [54]
	DCR: 23%		
	*Royal Marsden*	Consistent with ipilimumab clinical trials	Khattak et al., 2013 [55]
	DCR: 20%		
	*US EAP* 1- year OS: 34%	Consistent with wider EAP population	Lawrence et al., 2012 [50,51]
	*Multicentre retrospective analysis*		Luke et al., 2013 [56]
	DCR: 46%	Consistent with ipilimumab clinical trials	
Mucosal melanoma	*Italian EAP*		
	DCR: 36%	Safety profile similar to that in cutaneous melanoma	Del Vecchio et al., 2013 [57]
	1- year OS: 35%		
	*US EAP* 1- year OS: 32%	Consistent with wider EAP population	Lawrence et al., 2012 [50,51]
	Multicentre experience		Postow et al., 2013 [58]
	DCR: 27%	Consistent with ipilimumab clinical trials	
Brain metastases	*CA184-042 phase 2 trial (asymptomatic)*		
	DCR: 25%	Safety results consistent with those previously reported in clinical trials	Margolin et al., 2012 [59]
	1- year OS: 36%		
	2-year OS: 21%		
	*NIBIT-M1 phase 2 trial (+fotemustine; asymptomatic)*		
		DCR: 50%		
		1- year OS: 55%	AEs generally manageable and reversible	Di Giacomo et al., 2012 [60]
		2-year OS: 39%		Di Giacomo et al., 2013 [61]
	*Italian EAP* DCR: 27%	Safety results consistent with those previously reported in clinical trials	Queirolo et al., 2014 [62]	
	1- year OS: 20%			
	*US EAP* 1- year OS: 25%	Consistent with wider EAP population	Lawrence et al., 2012 [50,51]	
BRAF/NRAS-mutated melanoma	*Phase 2 study CA184-004*			
	(BRAF mutated vs BRAF wild-type)		Shahabi et al., 2012 [63]	
	DCR: 30% vs 35%			
	*NIBIT-M1 phase 2 trial*			
	*(+fotemustine; asymptomatic)*		Di Giacomo et al., 2013 [61]	
	(BRAF mutated vs BRAF wild-type)			
	DCR: 60% vs 46%			
	*Italian EAP*			
		(BRAF mutated vs BRAF wild-type)	Consistent regardless of BRAF and NRAS mutation status	Queirolo et al., 2014 [62]
	DCR: 38% vs 39%			
	1-year OS: 48% vs 39%			
	(NRAS mutated vs NRAS wild-type)			
	DCR: 57% vs 49%			
	1-year OS: 43% vs 40%			
	*4 institution retrospective analysis*			
	*(ipiliumab or tremelimumab)*			
	Similar median OS between patients with BRAF/NRAS-mutated and BRAF/NRAS wild-type melanoma; trend towards improved OS in wild-type population without prior BRAFi/MEKi treatment		Mangana et al., 2013 [46]	

AEs, adverse events; BRAFi, BRAF inhibitor; DCR, disease control rate; EAP, expanded access programme; I-OMEAP, ipilimumab-ocular melanoma expanded access program; MEKi, MEK inhibitor; NYU, New York University; OS, overall survival.

BRAF mutations are uncommon in patients with noncutaneous (uveal or mucosal) melanomas [64]; therefore, BRAF inhibitors may have limited utility in these patient populations. Although clinical trial data regarding the use of novel therapies in patients with noncutaneous melanoma are limited, data from EAPs suggest ipilimumab has a similar efficacy and safety profile in patients with advanced uveal or mucosal melanoma to that observed in cutaneous melanoma [50,51,53-55,57]. In EAP in Italy, the 1-year OS rates in patients with uveal or mucosal melanoma were 31% and 35%, respectively; these values are similar to those reported in the US EAP (34% and 32%, respectively) [50,53,57].

Subgroup analyses from the registrational phase 3 trial (MDX010-20) also suggest ipilimumab provides a consistent survival benefit across patient populations, including those with a historically poor prognosis (e.g. elevated lactate dehydrogenase or poor performance status) [2,65]. In addition, ipilimumab has demonstrated activity in elderly patients and patients with stable asymptomatic brain metastases, providing further support for the potential benefit with immunotherapy in a broad patient population [50,52,58,60,62]. With respect to the clinical activity of other immuno-oncology agents, in a phase 1 trial in patients with advanced solid tumours, objective response rates with nivolumab in NSCLC were similar across different patient groups and histology types [66].

Based on their mechanisms of action and available clinical data, immunotherapy agents may provide greatest clinical benefit if used as early as possible in the treatment paradigm when patients have a better prognosis. Furthermore, patients who eventually undergo disease progression after immunotherapy may have prolonged survival, which may allow the opportunity to receive and potentially benefit from subsequent lines of therapy. In MDX010-20, ipilimumab, with or without gp100, significantly improved median OS compared with gp100 alone despite similar estimates of median progression-free survival among the three treatment arms [2]. This may suggest that, even in some patients without an objective response or stable disease, immunotherapy can prolong survival by slowing the rate of disease progression.

Unlike targeted agents, immuno-oncology agents do not alter the nature of tumour cells, which could otherwise select for rapid disease kinetics. Therefore, initial treatment with immunotherapy does not compromise the ability of patients to respond to subsequent therapy with a BRAF inhibitor. Conversely, around 40% of patients with advanced melanoma who progress after BRAF inhibitor treatment undergo rapid disease progression and thus are unable to complete ipilimumab therapy. In a retrospective analysis of 34 patients with BRAF-mutated melanoma, among 28 patients who received a BRAF inhibitor followed by ipilimumab, 12 patients (43%) had rapid disease progression and were not able to complete ipilimumab treatment. Median OS for patients with rapid progression was 5.7 months, compared with 18.6 months for patients who were able to complete ipilimumab treatment. By comparison, none of the six patients who received ipilimumab followed by a BRAF inhibitor had rapid disease progression and all had disease control after subsequent treatment with a BRAF inhibitor [67]. Similar results were observed in the Italian EAP, whereby median OS was 9.9 and 14.5 months, respectively, for patients who received a BRAF inhibitor before or after ipilimumab treatment. Among 45 patients who received a BRAF inhibitor first, median OS from the end of BRAF inhibitor treatment was significantly longer in patients who were able to complete ipilimumab treatment compared with those who had rapid progression and were unable to complete ipilimumab therapy (12.7 versus 1.2 months; *P* < 0.001). With regard to optimal treatment sequencing, using immunotherapy first followed by targeted therapy in patients with more indolent disease may therefore offer the best chance of long-term survival [67-71].

Prior exposure to ipilimumab does not appear to affect outcomes to subsequent anti-PD1 antibody therapy. For example, in a phase 1 expansion study in 135 patients with advanced melanoma who received pembrolizumab, efficacy and safety in 48 patients who had received prior treatment with ipilimumab was similar to that observed in the 87 patients who were ipilimumab treatment-naïve [36].

Although typically used only as palliative therapy or in patients with CNS metastases, there is some evidence to suggest that administering RT after iplimumab may provide additional clinical benefit. For example, in a retrospective analysis of 21 patients who received locoregional RT after progressing on ipilimumab, 11 patients (52%) showed evidence of a systemic objective response or prolonged stable disease outside of the irradiated area [72]. This so-called ‘abscopal effect’ has also been observed in isolated patient cases when RT has been administered either before or after ipilimumab therapy, suggesting that RT and ipilimumab have potentially synergistic effects on antitumour immunity [73,74].

Ongoing research in oncology focuses on enhancing the proportion of patients who benefit from treatment with immunotherapy. As already eluded to, combining immunotherapies with other treatment modalities such as radiotherapy, chemotherapy or targeted agents could potentially lead to enhanced efficacy as these treatments may have immune-stimulatory properties. Increasing preclinical and clinical evidence suggests that these treatments may have additive effects when administered in combination with immunotherapy [60,73-76].

Given the numerous immune checkpoints that exist, combining immuno-oncology agents that target different checkpoint pathways is also an attractive therapeutic approach. Tumours exploit these pathways to turn off the immune response in different ways, either by decreasing T-cell proliferation or inactivating T cells at the tumour site [77]. For example, by inhibiting CTLA-4, ipilimumab promotes T-cell proliferation, increasing the number of activated T cells that can migrate to attack the tumour; conversely, anti-PD1 agents such as nivolumab counteract tumour defences specifically within the tumour microenvironment, reactivating T-cell activity and inducing tumour cell death [9,11]. The complementary roles of these two pathways in regulating adaptive immunity are supported by preclinical models in which simultaneous administration of anti-CTLA-4 and anti-PD1 antibodies resulted in enhanced antitumour activity compared with single agent treatments [78,79]. In a phase 1 combination study of ipilimumab and nivolumab in patients with advanced melanoma, 40% of patients treated with the concurrent combination regimen had objective responses and the 1-year OS rate was 82%; notably, responses with the combination were both rapid and deep [80,81]. This combination is being further evaluated versus ipilimumab or nivolumab monotherapy in a phase 3, randomised trial. Accumulating preclinical and clinical data also support the use of other investigational immunotherapy combinations such as nivolumab plus anti-LAG3 and ipilimumab plus GM-CSF [82,83].

Finally, biomarker discovery efforts may help to identify patients who are most likely to benefit from treatment with immunotherapy; immunological markers measured during treatment could also be useful as surrogate markers of clinical response. However, identification of immunotherapy biomarkers is challenging due to the complexity of interactions between the immune system and tumour cells, as well as difficulties in performing standardised immunological assays [84]. At present, no predictive biomarkers have been validated that can be used to guide patient selection for treatment with immunotherapy. Although there are some data to suggest that objective response rates with nivolumab, either as monotherapy or in combination with ipilimumab, are highest in patients with positive PDL1 expression in NSCLC and melanoma, the absence of this marker is not an indication that patients will fail to respond to treatment [85,86].

Given that immunotherapy appears to be effective in a broad patient population, the use of biomarkers to guide treatment selection may not be as relevant for immunotherapies as it is for targeted agents such as BRAF or MEK inhibitors. However, despite the lack of any definitive predictive biomarkers, several immunological parameters have been identified that may serve as early markers of response. For example, increases in absolute lymphocyte count and in the number of circulating T cells that express inducible T-cell costimulator during ipilimumab induction therapy have been found to be associated with higher response rates and/or improved survival outcomes [27,87-89]. Further prospective, controlled studies are needed to determine whether changes in these markers can be used to predict treatment effects.

## Conclusion

Over recent years, immunotherapy has increasingly been acknowledged as the fourth pillar of treatment in advanced cancer alongside surgery, radiotherapy, and chemotherapy, with ipilimumab in advanced melanoma serving as the model for proof of concept. Since the approval of ipilimumab in 2011, a number of post-approval issues of importance to practitioners have arisen, many of which are related to the mechanism of action of immuno-oncology agents. Among these is the potential for immunotherapy to show clinical activity in all subpopulations regardless of tumour genotype or histological subtype. Immunotherapy approaches may also allow the opportunity to slow disease progression and prolong survival even in patients with progressive disease. Another related issue which concerns clinicians is how to optimise the sequencing of treatment with ipilimumab and BRAF inhibitors, with increasing evidence to suggest that clinical benefit may be optimised by administering immunotherapy agents as early as possible in the treatment paradigm. In conclusion, immunotherapy agents may represent the future standards of care for various solid tumours or haematological malignancies, with the potential for providing a meaningful survival benefit. Various strategies combining immune checkpoint regulators with other complementary immunotherapies or different treatment modalities are under investigation to maximise treatment outcomes.

## Abbreviations

AJCC: American joint committee on cancer; CP: Carboplatin and paclitaxel; CTLA-4: cytotoxic T-lymphocyte antigen-4; EAP: Expanded access programme; GM-CSF: Granulocyte-macrophage colony-stimulating factor; irRC: Immune-related response criteria; mCRPC: Metastatic castration-resistant prostate cancer; mWHO: Modified World Health Organisation; NSCLC: Non-small cell lung cancer; OS: Overall survival; PD1: Programmed death 1; PDL2/PDL2: Programmed death ligand 1/2; PFS: Progression-free survival; RCC: Renal cell carcinoma; RT: Radiotherapy; TNM: Tumor-node-metastasis; Treg: Regulatory T cell; T-VEC: Talimogene laherparepvec; UICC: International union against cancer.

## Competing interests

PAA received research funding from Bristol-Myers Squibb, Roche-Genentech, and Ventana. He also has/had a consultant or advisory role for Bristol-Myers Squibb, Roche-Genentech, Merck Sharp & Dohme, GlaxoSmithKline Ventana, and Novartis. He received honoraria from Bristol-Myers Squibb, Roche-Genentech, and GlaxoSmithKline. FMM has no competing interest to declare.

## Author’s contribution

PAA and FMM drafted and approved the final manuscript.

## References

[B1] Lesterhuis WJ Haanen JB Punt CJ Cancer immunotherapy – revisited Nat Rev Drug Discov 2011 10 591 600 10.1038/nrd3500 21804596

[B2] Hodi FS O’Day SJ McDermott DF Weber RW Sosman JA Haanen JB Gonzalez R Robert C Schadendorf D Hassel JC Akerley W van den Eertwegh AJ Lutzky J Lorigan P Vaubel JM Linette GP Hogg D Ottensmeier CH Lebbé C Peschel C Quirt I Clark JI Wolchok JD Weber JS Tian J Yellin MJ Nichol GM Hoos A Urba WJ Improved survival with ipilimumab in patients with metastatic melanoma N Engl J Med 2010 363 711 723 10.1056/NEJMoa1003466 20525992 PMC3549297

[B3] Yervoy (ipilimumab) EU summary of product characteristics, Dec 2013 [http://www.ema.europa.eu/docs/en_GB/document_library/EPAR_-_Product_Information/human/002213/WC500109299.pdf]

[B4] Peggs KS Quezada SA Korman AJ Allison JP Principles and use of anti-CTLA4 antibody in human cancer immunotherapy Curr Opin Immunol 2006 18 206 213 10.1016/j.coi.2006.01.011 16464564

[B5] Chen L Files DB Molecular mechanisms of T cell co‑stimulation and co‑inhibition Nature Rev Immunol 2013 13 227 242 10.1038/nri3405 23470321 PMC3786574

[B6] Peggs KS Quezada SA Chambers CA Korman AJ Allison JP Blockade of CTLA-4 on both effector and regulatory T cell compartments contributes to the antitumor activity of anti–CTLA-4 antibodies J Exp Med 2009 206 1717 1725 10.1084/jem.20082492 19581407 PMC2722174

[B7] Wolchok JD Saenger Y The mechanism of anti-CTLA-4 activity and the negative regulation of T-cell activation Oncologist 2008 13 2 9 10.1634/theoncologist.13-S4-2 19001145

[B8] Blank CU The perspective of immunotherapy: new molecules and new mechanisms of action in immune modulation Curr Opin Oncol 2014 26 2 204 214 10.1097/CCO.0000000000000054 24424272

[B9] Mellman I Coukos G Dranoff G Cancer immunotherapy comes of age Nature 2011 480 481 489 10.1038/nature10673 PMC3967235 22193102

[B10] Pardoll DM The blockade of immune checkpoints in cancer immunotherapy Nat Rev Cancer 2012 22 252 264 22437870 10.1038/nrc3239 PMC4856023

[B11] Topalian SL Drake CG Pardoll DM Targeting the PD-1/B7-H1(PD-L1) pathway to activate antitumor immunity Curr Opin Immunol 2012 24 207 212 10.1016/j.coi.2011.12.009 22236695 PMC3319479

[B12] ClinicalTrials.gov: a registry and results database of publicly and privately supported clinical studies of human participants conducted around the world Clinicaltrials.gov, accessed March 2014

[B13] Dotti G Gottschalk S Savoldo B Brenner MK Design and development of therapies using chimeric antigen receptor-expressing T cells Immunol Rev 2014 257 1 107 126 10.1111/imr.12131 24329793 PMC3874724

[B14] Wu R Forget MA Chacon J Bernatchez C Haymaker C Chen JQ Hwu P Radvanyi LG Adoptive T-cell therapy using autologous tumor-infiltrating lymphocytes for metastatic melanoma: current status and future outlook Cancer J 2012 18 2 160 175 10.1097/PPO.0b013e31824d4465 22453018 PMC3315690

[B15] Lu YC Yao X Li YF El-Gamil M Dudley ME Yang JC Almeida JR Douek DC Samuels Y Rosenberg SA Robbins PF Mutated PPP1R3B is recognized by T cells used to treat a melanoma patient who experienced a durable complete tumor regression J Immunol 2013 190 12 6034 6042 10.4049/jimmunol.1202830 23690473 PMC3679246

[B16] Ledford H Cancer treatment: the killer within Nature 2014 508 7494 24 26 10.1038/508024a 24695297

[B17] Weiss SA Chandra S Pavlick AC Update on vaccines for high-risk melanoma Curr Treat Options Oncol 2014 Epub ahead of print 10.1007/s11864-014-0283-7 24788575

[B18] Galluzzi L Lugli E Cancer immunotherapy turns viral OncoImmunology 2013 2 e24801 e24802 10.4161/onci.24802 PMC3654608 23734338

[B19] Andtbacka RHI Collichio FA Amatruda T Senzer NN Chesney J Delman KA Spitler LE Puzanov I Doleman S Ye Y Vanderwalde AM Coffin R Kaufman H OPTiM: A randomized phase III trial of talimogene laherparepvec (T-VEC) versus subcutaneous (SC) granulocyte-macrophage colony-stimulating factor (GM-CSF) for the treatment (tx) of unresected stage IIIB/C and IV melanoma [abstract] J Clin Oncol 2013 31 suppl LBA9008

[B20] Kaufman H Andtbacka RHI Harrington K Collichio F Amatruda T Senzer N Chesney J Delman KA Vanderwalde AM Coffin R Secondary endpoints from OPTiM: A multicenter, randomized phase 3 trial of talimogene laherparepvec vs GM-CSF for the treatment of unresected stage IIIB/C and IV melanoma [abstract] Eur J Cancer 2013 49 suppl 2 3733

[B21] Karyampudi L Lamichhane P Scheid AD Kalli KR Shreeder B Krempski JW Behrens MD Knutson KL Accumulation of memory precursor CD8 T cells in regressing tumors following combination therapy with vaccine and anti-PD-1 antibody Cancer Res 2014 Epub ahead of print 10.1158/0008-5472.CAN-13-2564 PMC4313351 24728077

[B22] Angell H Galon J From the immune contexture to the Immunoscore: the role of prognostic and predictive immune markers in cancer Curr Opin Immunol 2013 25 261 267 10.1016/j.coi.2013.03.004 23579076

[B23] Wolchok JD Hoos A O’Day S Weber JS Hamid O Lebbé C Maio M Binder M Bohnsack O Nichol G Humphrey R Hodi FS Guidelines for the evaluation of immune therapy activity in solid tumors: immune-related response criteria Clin Cancer Res 2009 15 7412 7420 10.1158/1078-0432.CCR-09-1624 19934295

[B24] Ribas A Chmielowski B Glaspy JA Do we need a different set of response assessment criteria for tumor immunotherapy? Clin Cancer Res 2009 15 7116 7118 10.1158/1078-0432.CCR-09-2376 19934296

[B25] Topalian SL Sznol M Brahmer JR McDermott DF Smith DC Gettinger SN Taube JN Drake CG Pardoll DM Powderly JD Carvajal RD Sosman JA Atkins MB Antonia SJ Spigel DR Lawrence DP Kollia G Gupta AK Wigginton JM Hodi FS Nivolumab (anti-PD-1; BMS-936558; ONO-4538) in patients with advanced solid tumors: Survival and long-term safety in a phase I trial [abstract] J Clin Oncol 2013 31 suppl 3002

[B26] Ribas A Hersey P Middleton MR Gogas H Flaherty KT Sondak VK Kirkwood JM New challenges in endpoints for drug development in advanced melanoma Clin Cancer Res 2012 18 336 341 10.1158/1078-0432.CCR-11-2323 22142824 PMC3422891

[B27] Di Giacomo AM Calabrò L Danielli R Fonsatti E Bertocci E Pesce I Fazio C Cutaia O Giannarelli D Miracco C Biagioli M Altomonte M Maio M Long-term survival and immunological parameters in metastatic melanoma patients who responded to ipilimumab 10 mg/kg within an expanded access programme Cancer Immunol Immunother 2013 62 1021 1028 10.1007/s00262-013-1418-6 23591982 PMC11029072

[B28] Maio M Bondarenko I Robert C Thomas L Garbe C Testori A Lu H Chin K Wolchok JD Survival analysis with 5 years of follow-up in a phase III study of ipilimumab and dacarbazine in metastatic melanoma [abstract] Eur J Cancer 2013 49 suppl 2 3704

[B29] Prieto PA Yang JC Sherry RM Hughes MS Kammula US White DE Levy CL Rosenberg SA Phan GQ CTLA-4 blockade with ipilimumab: long-term follow-up of 177 patients with metastatic melanoma Clin Cancer Res 2012 18 7 2039 2047 10.1158/1078-0432.CCR-11-1823 22271879 PMC3319861

[B30] Robert C Thomas L Bondarenko I O'Day S JW MD Garbe C Lebbe C Baurain JF Testori A Grob JJ Davidson N Richards J Maio M Hauschild A Miller WH Jr Gascon P Lotem M Harmankaya K Ibrahim R Francis S Chen TT Humphrey R Hoos A Wolchok JD Ipilimumab plus dacarbazine for previously untreated metastatic melanoma N Engl J Med 2011 364 2517 2526 10.1056/NEJMoa1104621 21639810

[B31] Wolchok JD Weber JS Maio M Neyns B Harmankaya K Chin K Cykowski L de Pril V Humphrey R Lebbé C Four-year survival rates for patients with metastatic melanoma who received ipilimumab in phase II clinical trials Ann Oncol 2013 24 2174 2180 10.1093/annonc/mdt161 23666915 PMC4081656

[B32] Kwon ED Drake CG Scher HI Fizazi K Bossi A van den Eertwegh AJ Krainer M Houede N Santos R Mahammedi H Ng S Maio M Franke FA Sundar S Agarwal N Bergman AM Ciuleanu TE Korbenfeld E Sengeløv L Hansen S Logothetis C Beer TM McHenry MB Gagnier P Liu D Gerritsen WR for the CA184-043 Investigators Ipilimumab versus placebo after radiotherapy in patients with metastatic castration-resistant prostate cancer that had progressed after docetaxel chemotherapy (CA184-043): a multicentre, randomised, double-blind, phase 3 trial Lancet Oncol 2014 15 700 712 10.1016/S1470-2045(14)70189-5 24831977 PMC4418935

[B33] Schadendorf D Hodi FS Robert C Weber JS Margolin K Hamid O Chen TT Berman DM Wolchok JD Pooled analysis of long-term survival data from phase II and phase III trials of ipilimumab in metastatic or locally advanced, unresectable melanoma [abstract] Eur J Cancer 2013 49 suppl 2 24LBA 10.1200/JCO.2014.56.2736 PMC5089162 25667295

[B34] Hodi FS Topalian SL Brahmer JR McDermott DF Smith DC Gettinger S Taube JM Pardoll DM Wigginton JM Sznol M Survival and long-term safety in patients (pts) with advanced solid tumors receiving nivolumab (anti-PD-1; BMS-936558; ONO-4538) [abstract] Eur J Cancer 2013 49 suppl 2 880

[B35] Topalian SL Sznol M McDermott DF Kluger HM Carvajal RD Sharfman WH Brahmer JR Lawrence DP Atkins MB Powderly JD Leming PD Lipson EJ Puzanov I Smith DC Taube JM Wigginton JM Kollia GD Gupta A Pardoll DM Sosman JA Hodi FS Survival, durable tumor remission, and long-term safety in patients with advanced melanoma receiving nivolumab J Clin Oncol 2014 Epub ahead of print 10.1200/JCO.2013.53.0105 PMC4811023 24590637

[B36] Hamid O Robert C Daud A Hodi FS Hwu WJ Kefford R Wolchok JD Hersey P Joseph RW Weber JS Dronca R Gangadhar TC Patnaik A Zarour H Joshua AM Gergich K Elassaiss-Schaap J Algazi A Mateus C Boasberg P Tumeh PC Chmielowski B Ebbinghaus SW Li XN Kang SP Ribas A Safety and tumor responses with lambrolizumab (anti-PD-1) in melanoma N Engl J Med 2013 369 134 144 10.1056/NEJMoa1305133 23724846 PMC4126516

[B37] Brignone C Escudier B Grygar C Marcu M Triebel F A phase I pharmacokinetic and biological correlative study of IMP321, a novel MHC class II agonist, in patients with advanced renal cell carcinoma Clin Cancer Res 2009 15 6225 6231 10.1158/1078-0432.CCR-09-0068 19755389

[B38] Lynch TJ Bondarenko I Luft A Serwatowski P Barlesi F Chacko R Sebastian M Neal J Lu H Cuillerot JM Reck M Ipilimumab in combination with paclitaxel and carboplatin as first-line treatment in stage IIIB/IV non-small-cell lung cancer: results from a randomized, double-blind, multicenter phase II study J Clin Oncol 2012 30 2046 2054 10.1200/JCO.2011.38.4032 22547592

[B39] Reck M Bondarenko I Luft A Serwatowski P Barlesi F Chacko R Sebastian M Lu H Cuillerot JM Lynch TJ Ipilimumab in combination with paclitaxel and carboplatin as first-line therapy in extensive-disease-small-cell lung cancer: results from a randomized, double-blind, multicenter phase 2 trial Ann Oncol 2013 24 75 83 10.1093/annonc/mds213 22858559

[B40] Slovin SF Higano CS Hamid O Tejwani S Harzstark A Alumkal JJ Scher HI Chin K Gagnier P McHenry MB Beer TM Ipilimumab alone or in combination with radiotherapy in metastatic castration-resistant prostate cancer: results from an open-label, multicenter phase I/II study Ann Oncol 2013 24 1813 1821 10.1093/annonc/mdt107 23535954 PMC3707423

[B41] van den Eertwegh AJ Versluis J van den Berg HP Santegoets SJ van Moorselaar RJ van der Sluis TM Gall HE Harding TC Jooss K Lowy I Pinedo HM Scheper RJ Stam AG von Blomberg BM de Gruijl TD Hege K Sacks N Gerritsen WR Combined immunotherapy with granulocyte-macrophage colony-stimulating factor-transduced allogeneic prostate cancer cells and ipilimumab in patients with metastatic castration-resistant prostate cancer: a phase 1 dose-escalation trial Lancet Oncol 2012 13 509 517 10.1016/S1470-2045(12)70007-4 22326922

[B42] Brahmer JR Tykodi SS Chow LQ Hwu WJ Topalian SL Hwu P Drake CG Camacho LH Kauh J Odunsi K Pitot HC Hamid O Bhatia S Martins R Eaton K Chen S Salay TM Alaparthy S Grosso JF Korman AJ Parker SM Agrawal S Goldberg SM Pardoll DM Gupta A Wigginton JM Safety and activity of anti-PD-L1 antibody in patients with advanced cancer N Engl J Med 2012 366 26 2455 2465 10.1056/NEJMoa1200694 22658128 PMC3563263

[B43] Carnahan J Beltran PJ Babij C Le Q Rose MJ Vonderfecht S Kim JL Smith AL Nagapudi K Broome MA Fernando M Kha H Belmontes B Radinsky R Kendall R Burgess TL Selective and potent Raf inhibitors paradoxically stimulate normal cell proliferation and tumor growth Mol Cancer Ther 2010 9 2399 2410 10.1158/1535-7163.MCT-10-0181 20663930

[B44] Tafinlar (dabrafenib) EU summary of product characteristics, 2013 [http://www.ema.europa.eu/docs/en_GB/document_library/EPAR_-_Product_Information/human/002604/WC500149671.pdf]

[B45] Zelboraf (vemurafenib) EU summary of product characteristics, 2014 [http://www.ema.europa.eu/docs/en_GB/document_library/EPAR_-_Product_Information/human/002409/WC500124317.pdf]

[B46] Mangana J Goldinger SM Schindler K Rozati S Frauchiger AL Rechsteiner M Moch H Romano E Kaehler KC Michielin O Hauschild A Hoeller C Dummer R Analysis of BRAF and NRAS mutation status in advanced melanoma patients treated with anti-CTLA-4 antibodies: association with overall survival? [abstract] J Clin Oncol 2013 31 suppl 9025 10.1371/journal.pone.0139438 PMC4591284 26426340

[B47] Ascierto PA Simeone E Chiarion Sileni V Pigozzo J Maio M Altomonte M Del Vecchio M Di Guardo L Marchetti P Ridolfi R Cognetti F Testori A Bernengo MG Guida M Marconcini R Mandalà M Cimminiello C Rinaldi G Aglietta M Queirolo P Clinical experience with ipilimumab 3 mg/kg: Real-world efficacy and safety data from an expanded access programme cohort J Transl Med 2014 in press 10.1186/1479-5876-12-116 PMC4030525 24885479

[B48] Chiarion Sileni V Pigozzo J Ascierto PA Grimaldi AM Maio M Di Guardo L Marchetti P de Rosa F Nuzzo C Testori A Cocorocchio E Bernengo MG Guida M Marconcini R Merelli B Parmiani G Rinaldi G Aglietta M Grosso M Queirolo P Efficacy and safety of ipilimumab in elderly patients with pretreated advanced melanoma treated at Italian centres through the Expanded Access Programme J Exp Clin Cancer Res 2014 33 30 10.1186/1756-9966-33-30 24708900 PMC3996509

[B49] Lopez Martin JA Gonzalez Cao M Sereno M Mayordomo J Hidalgo M Campos B Cumplido D Zambrana F Medina J Berrocal A Ipilimumab in older patients: Spanish melanoma multidisciplinary group (GEM) experience in the expanded access programme [abstract] Ann Oncol 2012 23 suppl 9 3233

[B50] Lawrence D McDermott D Hamid O Weber J Wolchok J Richards J Minor D Pavlick A Sznol M Hwu P Urba W Amin A Bennett K Michener T Balogh A Hodi FS Treatment of Patients (pts) With Stage III or IV Melanoma on an Ipilimumab (Ipi) Expanded Access Program (EAP): Results for 3 mg/kg Cohort 2012 Hollywood, USA: Presented at Society for Melanoma Research (SMR) Congress

[B51] Lawrence D McDermott D Hamid O Weber J Wolchok J Richards J Amin A Bennett K Balogh A Hodi FS Ipilimumab (IPI) Expanded Access Program (EAP) for patients (pts) with Stage III/IV melanoma: safety data by subgroups [abstract] Ann Oncol 2012 23 suppl 9 1129P

[B52] Chandra S Madden KM Kannan R Pavlick AC Evaluating the safety of anti-CTLA-4 therapy in elderly patients with unresectable melanoma [abstract] J Clin Oncol 2013 31 suppl 9063

[B53] Maio M Danielli R Chiarion-Sileni V Pigozzo J Parmiani G Ridolfi R De Rosa F Del Vecchio M Di Guardo L Queirolo P Picasso V Marchetti P De Galitiis F Mandalà M Guida M Simeone E Ascierto PA Efficacy and safety of ipilimumab in patients with pre-treated, uveal melanoma Ann Oncol 2013 24 2911 2915 10.1093/annonc/mdt376 24067719

[B54] Danielli R Ridolfi R Chiarion-Sileni V Queirolo P Testori A Plummer R Boitano M Calabrò L Rossi CD Giacomo AM Ferrucci PF Ridolfi L Altomonte M Miracco C Balestrazzi A Maio M Ipilimumab in pretreated patients with metastatic uveal melanoma: safety and clinical efficacy Cancer Immunol Immunother 2012 61 41 48 10.1007/s00262-011-1089-0 21833591 PMC11028946

[B55] Khattak MA Fisher R Hughes P Gore M Larkin J Ipilimumab activity in advanced uveal melanoma Melanoma Res 2013 23 79 81 10.1097/CMR.0b013e32835b554f 23211837

[B56] Luke JJ Callahan MK Postow MA Romano E Ramaiya N Bluth M Giobbie-Hurder A Lawrence DP Ibrahim N Ott PA Flaherty KT Sullivan RJ Harding JJ D'Angelo S Dickson M Schwartz GK Chapman PB Wolchok JD Hodi FS Carvajal RD Clinical activity of ipilimumab for metastatic uveal melanoma: a retrospective review of the Dana-Farber cancer institute, Massachusetts general hospital, memorial Sloan-Kettering cancer center, and university hospital of Lausanne experience Cancer 2013 119 3687 3695 23913718 10.1002/cncr.28282 PMC3986037

[B57] Del Vecchio M Di Guardo L Ascierto PA Grimaldi AM Sileni VC Pigozzo J Ferraresi V Nuzzo C Rinaldi G Testori A Ferrucci PF Marchetti P De Galitiis F Queirolo P Tornari E Marconcini R Calabrò L Maio M Efficacy and safety of ipilimumab 3 mg/kg in patients with pretreated, metastatic, mucosal melanoma Eur J Cancer 2014 50 121 127 10.1016/j.ejca.2013.09.007 24100024

[B58] Postow MA Luke JJ Bluth MJ Ramaiya N Panageas KS Lawrence DP Ibrahim N Flaherty KT Sullivan RJ Ott PA Callahan MK Harding JJ D'Angelo SP Dickson MA Schwartz GK Chapman PB Gnjatic S Wolchok JD Hodi FS Carvajal RD Ipilimumab for patients with advanced mucosal melanoma Oncologist 2013 18 726 732 10.1634/theoncologist.2012-0464 23716015 PMC4063400

[B59] Margolin K Ernstoff MS Hamid O Lawrence D McDermott D Puzanov I Wolchok JD Clark JI Sznol M Logan TF Richards J Michener T Balogh A Heller KN Hodi FS Ipilimumab in patients with melanoma and brain metastases: an open-label, phase 2 trial Lancet Oncol 2012 13 459 465 10.1016/S1470-2045(12)70090-6 22456429

[B60] Di Giacomo AM Ascierto PA Pilla L Santinami M Ferrucci PF Giannarelli D Marasco A Rivoltini L Simeone E Nicoletti SV Fonsatti E Annesi D Queirolo P Testori A Ridolfi R Parmiani G Maio M Ipilimumab and fotemustine in patients with advanced melanoma (NIBIT-M1): an open-label, single-arm phase 2 trial Lancet Oncol 2012 13 879 886 10.1016/S1470-2045(12)70324-8 22894884

[B61] Di Giacomo AM Ascierto PA Queirolo P Pilla L Ridolfi R Santinami M Testori A Giannarelli D Parmiani G Maio M The Italian Network for Tumor Biotherapy (NIBIT)-M1 study: 2-years survival update for metastatic melanoma patients treated with ipilimumab in combination with fotemustine [abstract] Eur J Cancer 2013 49 suppl 2 3740

[B62] Queirolo P Spagnolo F Ascierto PA Simeone E Marchetti P Scoppola A Del Vecchio M Di Guardo L Maio M Di Giacomo AM Antonuzzo A Cognetti F Ferraresi V Ridolfi L Guidoboni M Guida M Pigozzo J Chiarion Sileni V Efficacy and safety of ipilimumab in patients with advanced melanoma and brain metastases J Neurooncol 2014 118 109 116 10.1007/s11060-014-1400-y 24532241 PMC4023079

[B63] Shahabi V Whitney G Hamid O Schmidt H Chasalow SD Alaparthy S Jackson JR Assessment of association between BRAF-V600E mutation status in melanomas and clinical response to ipilimumab Cancer Immunol Immunother 2012 61 733 737 10.1007/s00262-012-1227-3 22382362 PMC11029315

[B64] Edwards RH Ward MR Wu H Absence of BRAF mutations in UV-protected mucosal melanomas J Med Genet 2004 41 270 272 10.1136/jmg.2003.016667 15060100 PMC1735752

[B65] Lebbé C McDermott DF Robert C Lorigan P Ottensmeier CH Wolchok J Garbe C Messina M Hoos A Weber JS Ipilimumab improves survival in previously treated advanced melanoma patients with poor prognostic factors: subgroup analysis from a phase III trial [abstract] Ann Oncol 2010 21 suppl 8 13240

[B66] Gettinger SN Horn L Antonia SJ Spigel DR Gandhi L Sequist LV Sankar V Ahlers CM Wigginton JM Kollia G Gupta A Brahmer JR Efficacy of nivolumab (anti-PD-1; BMS-936558; ono-4538) in patients with previously treated advanced non-small cell lung cancer (NSCLC): subpopulation response analysis in a phase 1 trial [abstract] J Thorac Oncol 2013 8 suppl 2 2.11 038

[B67] Ascierto PA Simeone E Giannarelli D Grimaldi AM Romano A Mozzillo N Sequencing of BRAF inhibitors and ipilimumab in patients with metastatic melanoma: a possible algorithm for clinical use J Transl Med 2012 10 107 10.1186/1479-5876-10-107 22640478 PMC3464706

[B68] Ackerman A McDermott DF Lawrence DP Gunturi A Flaherty KT Giobbie-Hurder A Hodi FS Ibrahim N Atkins MB Cho DC Sullivan RJ Outcomes of patients with malignant melanoma treated with immunotherapy prior to or after vemurafenib [abstract] J Clin Oncol 2012 30 suppl 8569

[B69] Ascierto PA Simeone E Grimaldi AM Curvietto M Esposito A Palmieri G Mozzillo N Do BRAF inhibitors select for populations with different disease progression kinetics? J Transl Med 2013 11 61 10.1186/1479-5876-11-61 23497384 PMC3599508

[B70] Ascierto PA Simeone E Chiarion Sileni V Del Vecchio M Marchetti P Cappellini GC Ridolfi R de Rosa F Cognetti F Ferraresi V Testori A Queirolo P Bernengo MG Guida M Galli L Mandalà M Cimminiello C Rinaldi G Carnevale-Schianca F Maio M Sequential treatment with ipilimumab and BRAF inhibitors in patients with metastatic melanoma: data from the Italian cohort of the ipilimumab expanded access programme Cancer Invest 2014 32 144 149 10.3109/07357907.2014.885984 24484235

[B71] Jang S Atkins MB Which drug, and when, for patients with BRAF-mutant melanoma? Lancet Oncol 2013 14 e60 e69 10.1016/S1470-2045(12)70539-9 23369684

[B72] Grimaldi AM Simeone E Giannarelli D Muto P Falivene S Borzillo V Giugliano FM Sandomenico F Petrillo A Curvietto M Esposito A Paone M Palla M Palmieri G Caracò C Ciliberto G Mozzillo N Ascierto PA Abscopal effects of radiotherapy on advanced melanoma patients who progressed after ipilimumab immunotherapy Oncoimmunol 2014 in press 10.4161/onci.28780 PMC4106166 25083318

[B73] Postow MA Callahan MK Barker CA Yamada Y Yuan J Kitano S Mu Z Rasalan T Adamow M Ritter E Sedrak C Jungbluth AA Chua R Yang AS Roman RA Rosner S Benson B Allison JP Lesokhin AM Gnjatic S Wolchok JD Immunologic correlates of the abscopal effect in a patient with melanoma N Engl J Med 2012 366 925 931 10.1056/NEJMoa1112824 22397654 PMC3345206

[B74] Stamell E Wolchok JD Gnjatic S Lee NY Brownell I The abscopal effect associated with a systemic anti-melanoma immune response Int J Rad Onc Biol Phys 2013 85 293 295 10.1016/j.ijrobp.2012.03.017 PMC3415596 22560555

[B75] Hodge JW Ardiani A Farsaci Kwilas AR Gameiro SR The tipping point for combination therapy: cancer vaccines with radiation, chemotherapy, or targeted small molecule inhibitors Semin Oncol 2012 39 323 339 10.1053/j.seminoncol.2012.02.006 22595055 PMC3356994

[B76] Liu C Peng W Xu C Lou Y Zhang M Wargo JA Chen JQ Li HS Watowich SS Yang Y Tompers Frederick D Cooper ZA Mbofung RM Whittington M Flaherty KT Woodman SE Davies MA Radvanyi LG Overwijk WW Lizée G Hwu P BRAF inhibition increases tumor infiltration by T cells and enhances the antitumor activity of adoptive immunotherapy in mice Clin Cancer Res 2013 19 393 403 10.1158/1078-0432.CCR-12-1626 23204132 PMC4120472

[B77] Pardoll D Drake C Immunotherapy earns its spot in the ranks of cancer therapy J Exp Med 2012 209 201 209 10.1084/jem.20112275 22330682 PMC3280881

[B78] Curran MA Montalvo W Yagita H Allison JP PD-1 and CTLA-4 combination blockade expands infiltrating T cells and reduces regulatory T and myeloid cells within B16 melanoma tumors Proc Natl Acad Sci U S A 2010 107 4275 4280 10.1073/pnas.0915174107 20160101 PMC2840093

[B79] Selby M Engelhardt J Lu LS Quigley M Wang C Chen B Korman AJ Antitumor activity of concurrent blockade of immune checkpoint molecules CTLA-4 and PD-1 in preclinical models [abstract] J Clin Oncol 2013 31 suppl 306123569323

[B80] Wolchok JD Kluger H Callahan MK Postow MA Rizvi NA Lesokhin AM Segal NH Ariyan CE Gordon RA Reed K Burke MM Caldwell A Kronenberg SA Agunwamba BU Zhang X Lowy I Inzunza HD Feely W Horak CE Hong Q Korman AJ Wigginton JM Gupta A Sznol M Nivolumab plus ipilimumab in advanced melanoma N Engl J Med 2013 369 122 133 10.1056/NEJMoa1302369 23724867 PMC5698004

[B81] Wolchok JD Kluger HM Callahan MK Postow MA Gordon RA Segal NH Rizvi NA Lesokhin AM Reed K Burke MM Caldwell A Kronenberg SA Agunwamba B Feely W Hong Q Horak CE Korman AJ Wigginton JM Gupta AK Sznol M Clinical activity and safety of nivolumab (anti-PD-1, BMS-936558, ONO-4538) in combination with ipilimumab in patients with advanced melanoma [abstract] J Clin Oncol 2013 31 suppl 9021

[B82] Woo SR Turnis ME Goldberg MV Bankoti J Selby M Nirschl CJ Bettini ML Gravano DM Vogel P Liu CL Tangsombatvisit S Grosso JF Netto G Smeltzer MP Chaux A Utz PJ Workman CJ Pardoll DM Korman AJ Drake CG Vignali DA Immune inhibitory molecules LAG-3 and PD-1 synergistically regulate T-cell function to promote tumoral immune escape Cancer Res 2012 72 917 927 10.1158/0008-5472.CAN-11-1620 22186141 PMC3288154

[B83] Hodi FS Lee SJ McDermott DF Rao UNM Butterfield LH Tarhini AA Leming PD Puzanov I Kirkwood JM Multicenter, randomized phase II trial of GM-CSF (GM) plus ipilimumab (Ipi) versus Ipi alone in metastatic melanoma: E1608 [abstract] J Clin Oncol 2013 31 suppl CRA9007

[B84] Butterfield LH Palucka AK Britten CM Dhodapkar MV Håkansson L Janetzki S Kawakami Y Kleen TO Lee PP Maccalli C Maecker HT Maino VC Maio M Malyguine A Masucci G Pawelec G Potter DM Rivoltini L Salazar LG Schendel DJ Slingluff CL Jr Song W Stroncek DF Tahara H Thurin M Trinchieri G van Der Burg SH Whiteside TL Wigginton JM Marincola F Recommendations from the iSBTc-SITC/FDA/NCI Workshop on Immunotherapy Biomarkers Clin Cancer Res 2011 17 3064 3076 10.1158/1078-0432.CCR-10-2234 21558394 PMC3096674

[B85] Callahan MK Horak CE Curran MA Hollman T Schaer DA Yuan J Lesokhin AM Kitano S Hong Q Ariyan CE Busam KJ Feely W Jure-Kunkel M Grosso J Simon JS Korman AJ Wigginton JM Gupta AK Sznol M Wolchok JD Peripheral and tumor immune correlates in patients with advanced melanoma treated with combination nivolumab (anti-PD-1, BMS-936558, ONO-4538) and ipilimumab [abstract] J Clin Oncol 2013 31 suppl 3003

[B86] Grosso JF Horak CE Inzunza D Cardona DM Simon JS Gupta AK Sankar V Park JS Kollia G Taube JM Anders R Jure-Kunkel M Novotny J Taylor CR Zhang X Phillips T Simmons P Cogswell J Association of tumor PD-L1 expression and immune biomarkers with clinical activity in patients (pts) with advanced solid tumors treated with nivolumab (anti-PD-1; BMS-936558; ONO-4538) [abstract] J Clin Oncol 2013 31 suppl 3016

[B87] Delyon J Mateus C Lefeuvre D Lanoy E Zitvogel L Chaput N Roy S Eggermont AM Routier E Robert C Experience in daily practice with ipilimumab for the treatment of patients with metastatic melanoma: an early increase in lymphocyte and eosinophil counts is associated with improved survival Ann Oncol 2013 24 1697 1703 10.1093/annonc/mdt027 23439861

[B88] Simeone E Gentilcore G Giannarelli D Grimaldi AM Caracò C Curvietto M Esposito A Paone M Palla M Cavalcanti E Sandomenico F Petrillo A Botti G Fulciniti F Palmieri G Queirolo P Marchetti P Ferraresi V Rinaldi G Pistillo MP Ciliberto G Mozzillo N Ascierto PA Immunological and biological changes during ipilimumab treatment and their potential correlation with clinical response and survival in patients with advanced melanoma Cancer Immunol Immunother 2014 Apr 3. [Epub ahead of print] 10.1007/s00262-014-1545-8 PMC11028686 24695951

[B89] Wilgenhof S Du Four S Vandenbroucke F Everaert H Salmon I Liénard D Marmol VD Neyns B Single-center experience with ipilimumab in an expanded access program for patients with pretreated advanced melanoma J Immunother 2013 36 215 222 10.1097/CJI.0b013e31828eed39 23502769

